# Comprehensive Analysis of Genome Rearrangements in Eight Human Malignant Tumor Tissues

**DOI:** 10.1371/journal.pone.0158995

**Published:** 2016-07-08

**Authors:** Stefanie Marczok, Birgit Bortz, Chong Wang, Heike Pospisil

**Affiliations:** University of Applied Sciences Wildau, High Performance Computing in Life Sciences, Institute for Applied Biosciences, Wildau, Germany; Ohio State University Medical Center, UNITED STATES

## Abstract

Carcinogenesis is a complex multifactorial, multistage process, but the precise mechanisms are not well understood. In this study, we performed a genome-wide analysis of the copy number variation (CNV), breakpoint region (BPR) and fragile sites in 2,737 tumor samples from eight tumor entities and in 432 normal samples. CNV detection and BPR identification revealed that BPRs tended to accumulate in specific genomic regions in tumor samples whereas being dispersed genome-wide in the normal samples. Hotspots were observed, at which segments with similar alteration in copy number were overlapped along with BPRs adjacently clustered. Evaluation of BPR occurrence frequency showed that at least one was detected in about and more than 15% of samples for each tumor entity while BPRs were maximal in 12% of the normal samples. 127 of 2,716 tumor-relevant BPRs (termed ‘common BPRs’) exhibited also a noticeable occurrence frequency in the normal samples. Colocalization assessment identified 20,077 CNV-affecting genes and 169 of these being known tumor-related genes. The most noteworthy genes are *KIAA0513* important for immunologic, synaptic and apoptotic signal pathways, intergenic non-coding RNA *RP11-115C21.2* possibly acting as oncogene or tumor suppressor by changing the structure of chromatin, and *ADAM32* likely importance in cancer cell proliferation and progression by ectodomain-shedding of diverse growth factors, and the well-known tumor suppressor gene *p53*. The BPR distributions indicate that CNV mutations are likely non-random in tumor genomes. The marked recurrence of BPRs at specific regions supports common progression mechanisms in tumors. The presence of hotspots together with common BPRs, despite its small group size, imply a relation between fragile sites and cancer-gene alteration. Our data further suggest that both protein-coding and non-coding genes possessing a range of biological functions might play a causative or functional role in tumor biology. This research enhances our understanding of the mechanisms for tumorigenesis and progression.

## Introduction

The incidence of tumor increases rapidly with aging [1, 2], but the tumorigenesis is probably caused mainly by genetic preloads, bad environmental conditions and lifestyle behaviors [1, 3]. Cells affected by somatic mutations develop the typical hallmarks of cancer: sustaining proliferative signaling, evading growth suppressors, activating invasion and metastasis, enabling replicative immortality, inducing angiogenesis and resisting cell death [4]. Unrepaired genetic variations have long been thought associated with carcinogenesis [4, 5]. One type of genomic aberrations is copy number variation (CNV) that leads to an altered number (fewer or more) of copies of a genomic region in comparison with a reference genome. CNV arises from diverse mechanisms including nonallelic homologous recombination (NAHR), nonhomologous end joining (NHEJ) and fork stalling and template switching/microhomology-mediated break-induced replication (FoSTeS/MMBIR) during replication or recombination [6–8].

Previous studies [6, 9–13] have detected an increased number of CNVs in human malignant tumors and found a functional correlation of CNVs with tumorigenesis. For example, Gratias et al. [9] reported small deletions in retinoblastomas and CNVs at chromosomal band 16q24 known to encompass among others gene *KIAA0513*. The *KIAA0513* gene has been postulated to play an important role in immunologic, synaptic and apoptotic signal pathways [10]. Yang et al. [11] observed an increase of overall CNV burden in familial colorectal cancer patients compared with healthy controls as well as a novel structural variation at 12p12.3, suggesting a contribution of the overall burden of CNVs to familial colorectal cancer risk. *TP53*, also known as cellular tumor antigen p53, is involved in growth suppression and apoptosis. Several studies [11, 14–18] identified a remarkable number of CNVs characteristic of *TP53*-related tumors in Li-Fraumeni syndrome, an autosomal dominantly inherited disorder characterized by a variety of early-onset tumors. CNV breakpoints, forming a boundary between two copy number altered regions, have gained increasing attention in the mechanistic studies of tumorigenesis [19]. Li et al. [20] have identified cancer-type-specific breakpoint hotspots with distinct genomic patterns and found that these hotspots are enriched with known cancer genes. In an earlier study [13], we detected an increasing number of CNVs during tumor progression and identified some distinct breakpoint regions (BPRs) arising more frequently than other BPRs (e.g. fragile sites) in mouse mammary tumor. Our findings indicate that higher numbers of copy number alterations lead to an increased cancer risk [13]. In constrast, a recent work of Stewart et al. [3] supposed that tumor inducing mutations occur by chance and are readily inducible at common fragile sites. In a more recent study, Tomasetti et al. [21] showed that only one third of cancer cases are attributable to environmental factors or inherited predispositions. The evidence suggests that random mutations in regulatory genomic regions during cell division and the faulty repair mechanisms play the predominant role in carcinogenesis. These findings raise intriguing question regarding causal factors leading to cancer and the predictability of malignant events.

In the present study, we analyzed the influence of CNVs, BPRs and fragile sites on cancer development. In doing so, we investigated copy number altered regions and BPRs in 2,737 tumor samples from eight different tumor entities as well as in 432 normal samples from several tissue types including brain, gastric, lung, ovarian, prostate and renal tissues. Additionally, we evaluated the occurrences of tumor entity-specific, cancer-specific and common BPRs that would serve as a helpful clue to the mechanisms of tumorigenesis and tumor progression.

## Materials and Methods

### Data sets

To undertake a comprehensive analysis, a large number of tumor samples from more than one tumor entity with a high resolution must be analyzed. So far, the best resolution of genomic data is provided by next-, second- or third-generation sequencing technologies; however, these methods are very expensive and time consuming. A much cheaper and widely used alternative is represented by SNP arrays with a pretty good ratio of physical coverage and evaluating speed [22]. Moreover, there exists a huge pool of freely available data.

**Malignant tumor data:** The tumor data used for the genome-wide identification of CNVs and BPRs were taken from the publicly accessible Gene Expression Omnibus (GEO) database from the National Center for Biotechnology Information (NCBI) [23]. Specifically, raw CEL files from the Genome-Wide Human SNP Array 6.0 were analyzed in this study. Totally, 2,737 malignant primary tumor samples from 8 different tumor entities were used, including 377 breast tumor, 189 colorectal tumor, 340 gastric tumor, 291 lung tumor, 1,104 pediatric medulloblastoma, 207 ovarian tumor, 120 prostate tumor and 109 renal tumor samples. For a detailed description of the whole data set see S1 Table.

**Reference data:** Reference data were retrieved from the International HapMap Project (Phase 3, Release #3) [24, 25]. 990 HapMap samples analyzed by the Genome-Wide Human SNP Array 6.0 were taken into account to build a reference set for comparison (S2 Table).

**Normal samples:** Additionally, 432 normal samples from the GEO database were included in the study: brain tissue—29 samples, gastric tissue—148 samples, lung tissue—62 samples, ovarian tissue—57 samples, prostate tissue—67 samples, renal tissue—69 samples. These samples serve as the standard set for verification of CNV tumor specificity (S3 Table for further details).

### Algorithms

The detection of CNVs and tumor entity-specific, cancer-specific and common BPRs using SNP signal intensities proceeded through three steps: (1) preprocessing of SNP array raw data, (2) calculation of the logarithmized ratio (log_2_ ratio) of the signal intensities of the tumor sample and the reference set for each SNP, and (3) segmentation [26, 27]. A pipeline was implemented to facilitate these tasks. The preprocessing was carried out using the software Affymetrix Power Tool (APT, Linux version 1.16.0) [28] and the subsequent steps were performed within the freely available software R (R version 3.0.2) [29]. The segmentation and subsequent detection of CNVs and BPRs were performed for each tumor sample separately. All segments are defined by the respective chromosome, the number of encompassed SNPs and the segmental mean signal intensity. Furthermore, the start and end position of each segment is given as the genomic position of the first and last SNP of the segment. For the determination of the genomic position of each SNP, we used human genome hg19/GRCh37 as a reference.

**Building the reference:** To identify potential genomic alterations we built a reference set derived from 990 HapMap samples. Firstly we used the APT software package for preprocessing the raw data. Then the signal intensities for both SNP alleles were added up and the average signal intensity for all reference samples was calculated.

**Preprocessing and calculation of signal intensities:** All samples were preprocessed separately by quantile normalisation and a background correction with the Birdseed v2 algorithm provided by APT [11, 30]. The default program settings were used in the current study. The overall signal intensity of each SNP per sample was obtained by allele summation afterwards [31].

**Segmentation:** To detect genomic alteration in malignant tumors, we determined the segmentation profile for each sample. This was carried out by calculation of the log_2_ ratio of the signal intensities of the tumor sample in relation to the reference intensities for each SNP [26, 32]. The chromosomal segmentation of adjacent SNPs with similar log_2_ ratio values for the 22 autosomes was calculated using the circular binary segmentation algorithm (CBS algorithm) introduced by Olshen et al. [33] after outlier detection and data smoothing (“smooth.CNA”) [13]. The Bioconductor package DNAcopy (version 1.32.0) implements the circular binary segmentation algorithm and was used by setting the significance level *α* to 0.001, the standard deviation *SD* to 0.5 “sd.undo”) and the minimal number of markers per segment (“min.width”) to 4.

**Identification of copy number altered genomic regions** The resulting segments (representing the respective continuous genomic regions) of all the tumor samples were then further analyzed. The average value for each segment was accorded to its SNP intensity. The SNP intensities for all the samples belonging to each tumor entity were averaged and a mean value for each chromosome was calculated. The difference between each actual SNP intensity value and the chromosomal mean represents an altered copy number. We defined genomic regions with copy number alteration as segments exhibiting a difference of ≥ 0.1 or ≤ -0.1, respectively.

**Determination of genes localized in regions with copy number alteration:** The results of the determined regions with copy number alteration were used to discover if any gene could be affected by deleted or amplified genomic regions. The examination was done using the BiomaRt package (version 2.24.0) [34] in R, which offers access to several data sources including HapMap, HGNC, Ensembl, InterPro and Reactome. In the present study, gene detection was performed with Ensembl (version GRCh37.p13/ release 82).

**Breakpoint detection:** To detect chromosomal BPRs, we considered the regions of adjacent segments whose segment mean difference was > 0.6 (corresponding to 1 copy number based on the log_2_ transformation). A potential BPR was defined as the genomic stretch between the last SNP position of a segment and the first SNP position of its successive adjacent segment. The actual breakpoint lies somewhere between these two genomic positions, but could not be detected exactly due to the layout of microarrays. For identification of specific BPRs, the number of detected BPRs was counted.

**Determination of the different BPR classes:** Taking into consideration the occurrence frequency of BPRs and whether a BPR appeared also in normal samples, we devided BPRs into four BPR recurrency classes: tumor entity-specific BPR (occurrence is ≥ 1% in exclusively one tumor entity with or without being found in healthy samples) (1), cancer-specific BPR (occurrence is ≥ 1% in more than 25% of the entities and in normal samples < 0.5%) (2) and common BPR (occurrence is ≥ 1% in ≥ 25% of the entities and ≥ 0.5% in normal samples) (3), and no cancer-specific BPR (occurrence are < 1% in all tumor entities) (4).

## Results

### Identified BPRs

For all 2,737 tumor samples from eight tumor entities, 64,720 different BPRs were identified, and 7,324 of them in more than one tumor entity. On average, the BPRs span 6,831 bp and the size ranges from 10 bp to 22,757,511 bp. 127 BPRs were detetected in more than 1% of all the samples, 47 BPRs in at least 2% (Table 1) and 10 BPRs in 5% or more of the tumor samples. Furthermore, 8,853 BPRs were found exclusively in the normal samples and 7,695 BPRs in both the tumor and the normal samples (S7 Fig). A list of all the identified BPRs is given in S4 Table.

**Table 1:** 47 Breakpoint regions (BPRs) determined in over 2% of all sample sets.

chr	SNP A	SNP B	breast	color.	gastr.	lung	medul.	ov.	pro.	ren.	cancer	normal
**1**	148,916,177	149,040,066	11	5	9	9	23	4	1	0	62	5
**2**	172,338,300	172,339,964	30	27	1	5	25	17	0	8	113	3
	172,345,151	172,348,021	17	27	0	4	18	16	0	8	90	3
	242,718,444	242,725,752	7	8	3	2	51	1	1	7	80	6
**4**	9,994,215	9,996,852	47	0	5	4	0	0	0	4	60	0
	9,997,801	10,001,833	52	0	25	10	0	0	0	4	91	0
	70,950,894	70,951,184	8	12	0	0	96	0	0	5	121	0
	70,951,806	70,953,579	8	12	0	0	96	0	0	5	121	0
**5**	4,443,854	4,445,976	1	0	63	6	0	0	0	0	70	4
**6**	29,850,274	29,871,636	8	3	20	10	31	14	4	2	92	15
	29,899,493	29,899,677	2	1	11	3	29	8	3	0	57	9
	67,048,629	67,049,406	9	24	9	8	13	1	0	5	69	2
	77,422,497	77,439,868	27	19	8	15	67	10	2	6	154	14
	77,452,270	77,452,804	15	15	7	11	26	5	0	1	80	6
	77,452,804	77,461,073	12	4	1	4	41	5	1	5	73	8
	78,962,626	78,979,398	20	15	12	7	108	8	1	5	176	12
	79,026,686	79,039,234	15	15	7	5	104	8	0	4	158	11
**8**	5,594,132	5,601,352	8	6	7	4	29	5	2	2	63	6
	6,104,977	6,107,427	6	30	4	3	15	11	0	9	78	1
	39,225,941	39,288,762	29	23	16	24	97	9	1	10	209	24
	39,397,732	39,398,022	28	21	16	24	97	10	1	10	207	22
	43,778,914	46,924,211	34	7	9	21	7	17	17	6	118	13
	137,677,896	137,681,619	8	5	2	6	29	3	2	4	59	10
**11**	9,241,448	9,250,359	4	2	1	5	32	2	0	12	58	4
	86,410,303	86,410,905	7	13	1	1	56	4	0	3	85	1
	86,412,698	86,412,880	7	13	10	3	59	3	0	3	98	1
**12**	33,299,791	33,302,438	11	7	4	10	32	5	1	2	72	10
	33,303,866	33,312,197	24	7	3	10	43	5	1	3	96	11
**14**	41,606,882	41,606,899	2	7	4	2	50	0	0	3	68	5
	41,653,977	41,670,102	2	7	4	2	50	0	0	3	68	6
**16**	70,842,097	70,854,381	58	0	1	3	20	1	0	0	83	1
	71,202,489	71,207,879	30	0	2	3	19	8	0	0	62	0
	85,082,710	85,091,864	1	9	1	1	172	3	0	11	198	1
	85,091,864	85,092,483	42	43	32	76	308	44	0	12	557	21
	85,092,748	85,092,892	37	52	33	74	480	47	0	23	746	22
**17**	18,917,513	18,917,915	7	3	1	2	111	0	0	0	124	0
	18,917,915	19,168,912	2	1	0	0	170	0	0	0	173	0
	44,162,597	44,165,803	12	7	4	3	50	2	3	8	89	5
	44,572,303	44,789,285	17	12	4	6	61	4	4	7	115	5
	54,158,456	54,163,047	10	0	5	0	40	1	1	2	59	6
	54,172,591	54,173,463	11	0	10	3	33	5	1	1	64	3
**18**	4,976,160	4,979,612	1	31	14	0	15	0	0	28	89	51
	4,989,683	4,990,804	1	22	8	0	23	0	3	22	79	49
**21**	20,346,687	20,347,871	4	12	0	0	43	0	0	7	66	0
	20,353,826	20,353,905	4	13	2	0	43	0	0	6	68	0
	23,655,764	23,655,900	0	1	34	35	7	11	4	0	92	29
	23,664,658	23,667,121	2	5	39	39	40	24	9	1	159	39

BPRs are provided with their related chromosome (chr), start (SNP A) and end (SNP B) positions, absolute occurrence probability in the tumor entities (breast, color., lung, gastr., medul., ov., pro., ren.), and the total occurrence over all tumor samples (cancer) and in healthy tissue samples (normal), respectively.

Abbreviations: color.—colorectal; gastr.—gastric; medul. -pediatric medulloblastoma; ov.—ovarian; pro.—prostate; ren.—renal

### Tumor entity-specific, cancer-specific and common BPRs

To identify tumor entity-specific, cancer-specific and common breakpoint patterns, the occurrence of BPRs in each tumor entity was counted and compared between the entities. The most recurrent BPRs (2,279) are tumor entity-specific. 230 of the cancer-specific BPRs could be repeatedly detected in 25-75% and 7 in more than 75% of the tumor entities. The common BPRs could be determined 207 times in over 25% of the tumor entities. In addition, 32 BPRs were found to exhibit a dinstinct entity specificity. The noticeable occurrence frequency BPRs (NOF-BPRs) occurred in at least 10% of all the samples of the individual entities.

**Common BPRs:** Two of common BPRs appeared very frequently in seven out of eight tumor entities: one localized on chromosome 16 between 85,091,864 bp and 85,092,483 bp and the other on the same chromosome from 85,092,748 bp to 85,092,892 bp. The former BPR exists in 9.41 up to 27.90% of all the samples for each entity, but only in 4.86% of all the normal samples. The latter even ranges from 9.71 to 43.48% per tumor entity and was found only in 22 out of 432 normal samples. Other two adjacent interesting BPRs on chromosome 8 (chr8: 39,225,941 bp to 39,288,762 bp and 39,397,732 bp to 39,398,022 bp) were found to occur in all the tumor entities with a frequency up to 12.17% (S4 Table).

**Cancer-specific BPRs:** One of two cancer-specific BPRs occurring in more than 75% of the tumor entities could be identified in chromosome 8 (6,104,977 bp to 6,107,427 bp) with a frequency up to 15.87%. Two BPRs were detected in chromosome 4 (9,994,215 bp to 9,996,852 bp and 9,997,801 bp to 10,001,833 bp) at a mean frequency of 4.75 and 7.06%, respectively, and each in 50% of the tumor entities (S4 Table).

**Tumor entity-specific BPRs:** However, most BPRs were found to occur frequently in only one tumor entity. For example, a BPR on chromosome 17 (18,917,915 bp to 19,168,912 bp) was detected in 15.40% of all the medulloblastoma samples, but in less than 0.6% of all the samples of the other individual tumor entities (S4 Table).

**Fig 1:**
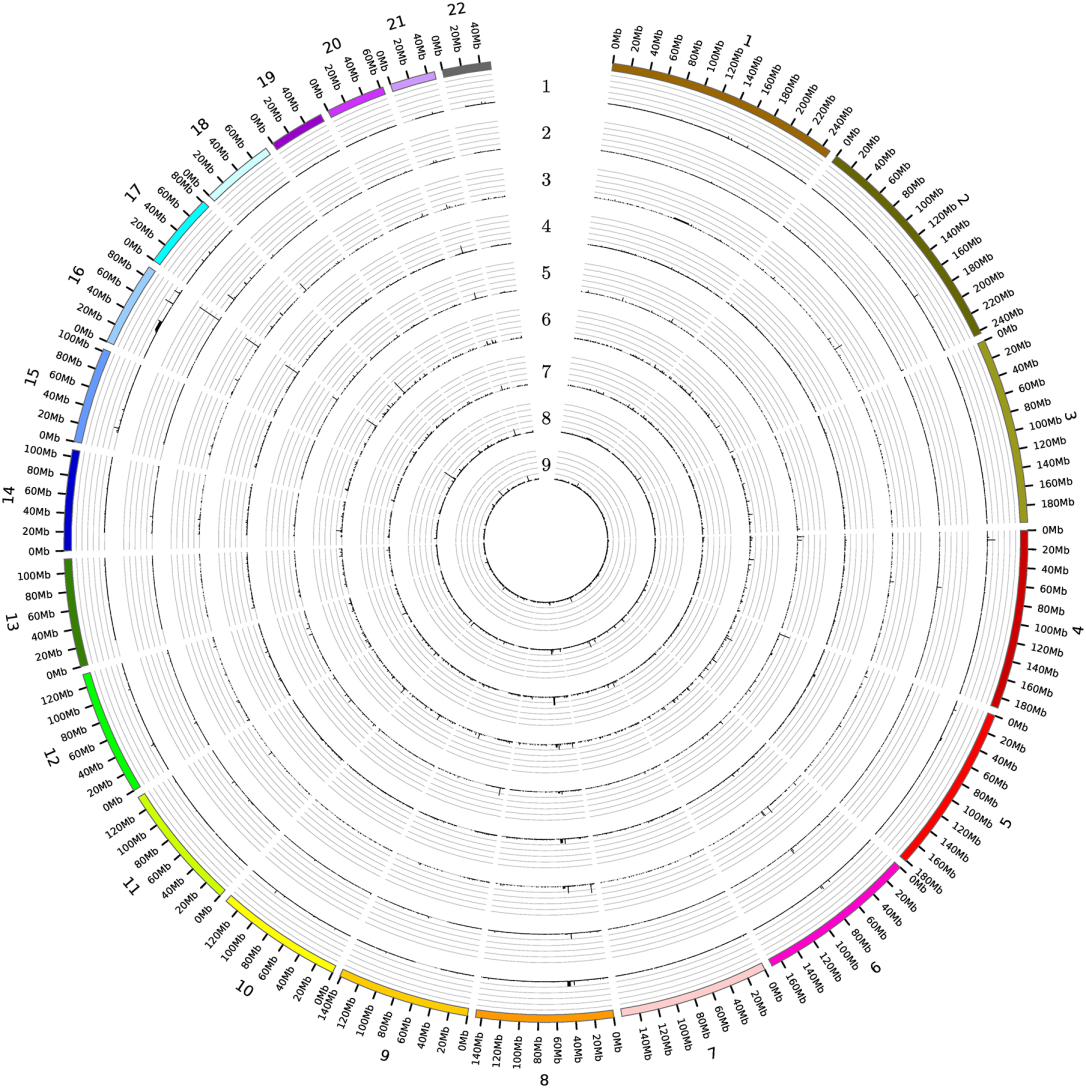
The occurrence of all the BPRs identified for the respective tumor entities and the healthy tissue samples over the full genome. Each circle corresponds to one tissue type (1- brain cancer (pediatric medulloblastoma), 2- breast cancer, 3- colorectal cancer, 4- gastric cancer, 5- lung cancer, 6- ovarian cancer, 7- prostate cancer, 8- renal cancer, 9- healthy tissue). Each gridline correlate to 10%.

**BPRs in healthy tissues:** Eight out of the 31 NOF-BPRs were also found in the normal samples with a frequency of 5% (common BPRs), but this amount is in all cases lower than those in the tumor samples. Only two BPRs from the NOF-BPRs set were found on chromosome 18 (4,976,160 bp to 4,979,612 bp and 4,989,683 bp to 4,990,804 bp) at a frequency greater than 10%, but more often detected in the renal and colorectal than the other tumor samples (Fig 1 and Table 2).

**Table 2:** 32 noticeable occurrence frequency breakpoint regions (NOF-BPRs), which was found in at least 10% of all samples in the single tumor entities and/or the healthy tissues.

chr	SNP A	SNP B	breast	color.	gastr.	lung	medul.	ov.	pro.	renal	normal	class
**2**	172,338,300	172,339,964	7.96	14.29	0.29	1.72	2.26	8.21	0.00	7.34	0.69	3
	172,345,151	172,348,021	4.51	14.29	0.00	1.37	1.63	7.73	0.00	7.34	0.69	3
**4**	3,027,493	3,027,897	0.00	0.00	11.47	0.00	0.00	0.00	0.00	1.83	1.39	3
	3,029,623	3,035,631	0.00	0.00	11.76	0.00	0.00	0.00	0.00	1.83	1.16	3
	9,994,215	9,996,852	12.47	0.00	1.47	1.37	0.00	0.00	0.00	3.67	0.00	2
	9,997,801	10,001,833	13.79	0.00	7.35	3.44	0.00	0.00	0.00	3.67	0.00	2
**5**	4,430,296	4,431,868	0.27	0.00	11.18	0.69	0.00	0.00	0.00	0.00	1.16	1
	4,443,854	4,445,976	0.27	0.00	18.53	2.06	0.00	0.00	0.00	0.00	0.93	3
	61,569,169	61,572,989	0.00	0.00	0.00	0.34	1.09	0.00	0.00	12.84	0.46	2
	126,152,745	126,158,200	0.27	0.00	0.00	1.37	1.54	0.00	0.00	10.09	1.16	3
**6**	67,048,629	67,049,406	2.39	12.70	2.65	2.75	1.18	0.48	0.00	4.59	0.46	2
	77,422,497	77,439,868	7.16	10.05	2.35	5.15	6.07	4.83	1.67	5.50	3.24	3
**7**	35,396,655	35,397,567	0.00	0.00	1.03	3.82	0.27	11.59	5.83	0.00	8.33	3
**8**	6,104,977	6,107,427	1.59	15.87	1.18	1.03	1.36	5.31	0.00	8.26	0.23	2
	39,225,941	39,288,762	7.69	12.17	4.71	8.25	8.79	4.35	0.83	9.17	5.56	3
	39,397,732	39,398,022	7.43	11.11	4.71	8.25	8.79	4.83	0.83	9.17	5.09	3
	43,778,914	46,924,211	9.02	3.70	2.65	7.22	0.63	8.21	14.17	5.50	3.01	3
**9**	9,337,599	9,338,146	0.27	0.53	13.53	0.34	0.00	0.00	0.00	0.00	0.46	1
	9,338,417	9,339,871	0.27	0.53	11.76	0.69	0.00	0.00	0.00	0.00	0.46	1
**10**	44,051,819	44,065,519	0.53	0.00	0.00	1.03	0.36	0.00	0.00	10.09	0.23	2
**11**	9,241,448	9,250,359	1.06	1.06	0.29	1.72	2.90	0.97	0.00	11.01	0.93	3
**14**	35,070,371	35,076,347	0.00	0.00	0.00	0.34	0.45	0.00	0.00	10.09	0.46	1
**16**	70,842,097	70,854,381	15.38	0.00	0.29	1.03	1.81	0.48	0.00	0.00	0.23	2
	85,082,710	85,091,864	0.27	4.76	0.29	0.34	15.58	1.45	0.00	10.09	0.23	2
	85,091,864	85,092,483	11.14	22.75	9.41	26.12	27.90	21.26	0.00	11.01	4.86	3
	85,092,748	85,092,892	9.81	27.51	9.71	25.43	43.48	22.71	0.00	21.10	5.09	3
**17**	18,917,513	18,917,915	1.86	1.59	0.29	0.69	10.05	0.00	0.00	0.00	0.00	2
	18,917,915	19,168,912	0.53	0.53	0.00	0.00	15.40	0.00	0.00	0.00	0.00	1
**18**	4,976,160	4,979,612	0.27	16.40	4.12	0.00	1.36	0.00	0.00	25.69	11.81	3
	4,989,683	4,990,804	0.27	11.64	2.35	0.00	2.08	0.00	2.50	20.18	11.34	3
**21**	23,655,764	23,655,900	0.00	0.53	10.00	12.03	0.63	5.31	3.33	0.00	6.71	3
	23,664,658	23,667,121	0.53	2.65	11.47	13.40	3.62	11.59	7.50	0.92	9.03	3

BPRs are provided with their related chromosome (chr), start (SNP A) and end (SNP B) positions, absolute occurrence probability in the tumor entities (breast, color., gastr., lung, medul., ov., pro., renal), and the total occurrence over all tumor samples (cancer) and in healthy tissue samples (normal), respectively, and the related classification of the BPRs (tumor entity-specific (1)—occurrence is ≥ 1% in exclusively one tumor entity with or without being found in healthy samples, cancer-specific (2)—occurrence is ≥ 1% in more than 25% of the entities and in normal samples < 0.5%, common (3)—occurrence is ≥ 1% in ≥ 25% of the entities and ≥ 0.5% in normal samples.

Abbreviations: color.—colorectal; gastr.—gastric; medul.—pediatric medulloblastoma; ov.—ovarian; pro.—prostate; ren.—renal

### Comparison of altered genomic regions within different tumor entities

To recognize a possible influence of genomic alterations on tumorigenesis and progression, the patterns of genomic regions featuring an alteration in copy number were examined to determine if they are overlapped with certain genes. Therefore, the average segment mean for each tumor entity was determined and the genomic regions with an abnormal number of copies were identified. We detected both cancer-specific and tumor entity-specific regions for altered copy number. One genomic region on chromosome 16 (85,091,864 bp to 85,092,748 bp) seemed to be deleted in seven out of eight tumor entities (S1 and S2 Figs). In six tumor entities (breast, colorectal, lung, ovarian, prostata and renal) the genomic region was between 161,222 bp to 39,397,732 bp on chromosome 8 deleted, in dependence of entity. Additionally, the genomic region from 6,689 bp to 18,917,915 bp on chromosome 17 is deleted in malignant tumor tissue by a factor of 0.6, whereas this segment was only slightly reduced (by a factor of 0.1) in breast, colorectal and ovarian tumor samples (S1 and S3 Figs). In all cases, the copy number is less altered in the normal tissue samples than in at least one type of tumor samples (S5–S13 Tables).

### Distribution of BPRs and segments of altered copy number in intragenic and intergenic regions

To mention the impact of BPRs and copy number variations we analyzed the frequency of BPRs and segments of copy number variations in intragenic and intergenic regions. The determined BPRs were nearly equally distributed over all eight tumor entities (only 5% difference) (S4 Fig). It is remarkable that the majority of BPRs is completely located within intragenic regions (25%) or within regions overlapping genes and non-coding segments (35-40%) of the human genome. However, the fact that nearly one third of the BPRs occupies intergenic regions indicates altered DNA structures within regulatory or functionally important regions as well as in regions of unknown functionality. The number of affected intergenic regions is very high in breast, gastrointestinal and renal tissues. (S4 and S5 Figs).

### Affected genes in regions of altered copy number

**Fig 2:**
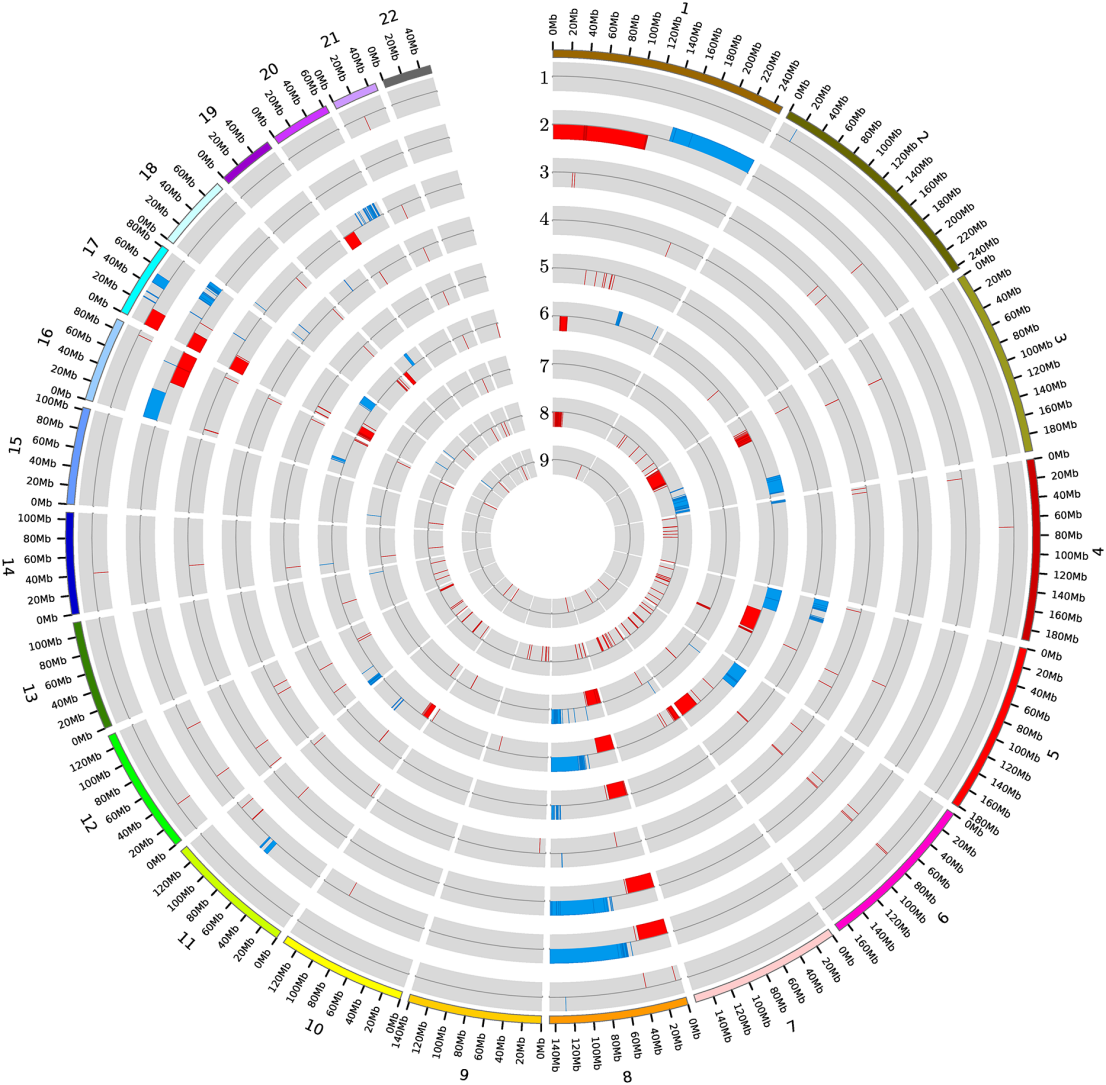
All segments with altered copy number over the full genome for the respective tumorentities and the healthy tissue samples. Each circle corresponds to one tissue type (1- brain cancer red(pediatric medulloblastoma), 2- breast cancer, 3- colorectal cancer, 4- gastric cancer, 5- lung cancer, 6- ovarian cancer, 7- prostate cancer, 8- renal cancer, 9- healthy tissues). Alterations are shown with red inner bar for a loss (deletion) and blue outer bar for a gain (amplification). The bars are localized to the respective genomic positions.

To identify genes localized within the copy number altered regions, 564 (mostly short) segments with continuous and concordant SNP signal intensity alterations were examined. Most of these segments (147) were derived from the ovarian tumor samples and the fewest (16) from the brain (pediatric medulloblastoma) tumor samples. By contrast, only 10 were found in the normal samples. On chromosome 8, longer genomic regions were affected in five different tumor entities, but only very short segments in the normal tissues. In renal tumor a remarkably increased number (127) of short (mostly deleted) regions could be observed. It is noticeable that in breast cancer the p-arm of chromosome 1 was mainly amplified and the q-arm almost completely deleted (Fig 2).

In all the eight tumor entities 20,062 genes within copy number altered regions were identified and 169 of them are described as tumor-associated. The majority of genes was found in breast and ovarian cancer (10,979 and 9,982, respectively) and the same holds true for tumor-associated genes (99 and 86, respectively). The fewest number of impacted genes and tumor-associated genes was encounted in gastric cancer with 66 and 6 genes, respectively. Only seven affected genes were found in the normal samples, but to the best of our knowledge none are associated with cancer (S6 Fig and S16 Table). Only one genomic region on chromosome 8, where the *ADAM* (a disintegrin and metalloproteinase) *32* gene is located, was found to be affected by copy number alteration in all the tumor entities as well as in the normal samples. In seven out of eight tumor entities, the gene *KIAA0513* was found to be concerned. 12 regions on chromosome 8 and one stretch on chromosome 21 were repeatedly found in six tumor entities. The same 838 gene regions were affected in five tumor entities. 826 of them are located on chromosome 8 and two on chromosome 17 (S14 and S15 Tables).

## Discussion

### Comparison of BPRs and copy number altered segments between tumor and normal samples

The genome-wide analysis show that the number of the identified BPRs ascertained together with the regions subject to copy number changes in tumor is approximately equal to the number in healthy genomes. But the occurrence of certain BPRs is higher in the tumor entities, while the distribution of BPRs, detected in healthy tissues, are nearly homogeneously distributed over the genome. For the copy number altered segments, the single tumor entities show a higher altered copy number than the healthy tissue. These results are in line with those of previous studies [11, 35]. On average, 70 segments per tumor entity were detected and only 10 in the normal samples. The equal number of BPRs in normal tissues in comparison with tumor samples supports the idea that genomic variations, especially CNVs, likely amount to 4.8-9.5% in healthy genomes [36]. In addition to the lower appearance of copy number altered segments in the normal samples we identified within those segments a very low number of affected genes. These affected genes would probably not alter the phenotypic outcome [36] and these are not known as being cancer-associated.

### Comparison of identified BPRs and copy number altered segments between different tumor entities

In the current study, many of the BPRs and copy number altered segments could be repeatedly found in multiple tumor samples and entities. To further investigate whether BPRs and segments with alteration in copy number are tumor entity-specific, cancer-specific or common genomic alterations, we evaluated the differences of these alterations between the eight tumor entities. We were able to find seemingly existing patterns in the genome-wide detected alterations as well as BPRs.

**Tumor entity-specific BPR:** By comparison of three cancer relevant classes, we found that most of the BPRs are tumor entity-specific. Each tumor entity exhibited an individual BPR-pattern to a certain extent, possibly indicating that tumorigenesis and tumor progression are partially due to individual genomic variations. A possible explanation for this might be the distinct differentiation of cells from various tissue sources. The difference in the activation of genomic regions across multiple tissue types could induce varying replication frequencies between different types of tissues [37]. For example, the probability of the incidence of a malignant heart tumor is very low, because the cardiomyocytes are postmitotic cells [38]. Consequently, the differentiation of a cell could have a crucial influence on the individual BPR-patterns and therefore also on the tumorigenesis and tumor progression.

**Cancer-specific BPR:** The cancer-specific BPR class is in the second place among the detected BPRs. The cancer-specific BPRs appeared only in the tumor and not in the healthy genomes. This class could be substantial for clarification of the common cancer risk factors associated with certain genomic positions, which promote the tumorigenesis.

**Common BPR:** The common BPR class was the least frequently occurring class found in this study. The BPRs and copy number altered segments identified in the tumor samples were also partly detected in the normal samples. Thereby, it is noticeable that the probability of the occurrence of the BPRs were in at least one tumor entity higher than in the normal samples. These results support the hypothesis relating to fragile sites that the genome at certain sites is unstable in contrast to other genomic positions [39]. The lower stability gives rise to a higher fragility for DNA breakage at those sites [40]. This supports our assumption of the increase in fragmentation of the genome during the tumor progression [13]. Because of the high frequency of replication of cancer cells, the probability of further breaks in those areas is very high. Such an event might account for the increasing occurrence of the common BPRs in the particular tumor entities. Similar findings were also reported in the literature [41].

### Presence of hotspot-regions

In this research, we observed an increased occurrence of several adjacent BPRs by comparison of the detected structural alterations. Also, we noted an overlapping of segments with similar copy number alterations, referred to as “hotspot-areas”. The presence of these areas are likely due to either technical or biological conditions. The technical reason might be the resolution and the layout of the detection method (SNP 6.0 Datasheet) [42]. These regions could also be exhibit a high gene activity with the result that the DNA strand is longer unwind. Within that areas the DNA cut randomly easier because of a minor stability [40]. On the other hand, stochastic effects related to DNA replications may contribute to cancer incidence [21]. Taken together, it is imaginable that the breakage would occur randomly. But in a few but important regions, they frequently arise due to genomic instability.

### Importance of the NOF-BPRs

To illustrate the importance of the NOF set of BPRs, we assessed the colocalization of these BPRs, the associated CNV segments and the potentially involved genes. Thereby, we found that these variants were related to both protein-coding and non-coding genes (e.g. lncRNA). About 0.8% of the identified genes are related to cancer. Most of the 31 NOF-BPRs fall into the common class (55%). Specifically, the most frequently detected BPR is located within the protein-coding gene *KIAA0513* (chr16: 85,061,374 bp to 85,127,836 bp). It is well known that this gene plays an important role in immunologic, synaptic and apoptotic signal pathways. Thus, a deletion within the gene, as we detected in 7 of 8 tumor entities and the normal samples, could disturb the gene expression and induce a loss of function as a signal molecule in apoptosis [10], consequently promoting the tumorigenesis.

Two other interesting common BPRs were found on chromosome 8. Between these regions the *ADAM32* gene is located (chr8: 39,308,564 bp to 39,380,371 bp). Additionally, in all 8 tumor entities and the normal samples there was a deletion within this gene. Previous studies [43–45] have reported that members of the ADAM family of proteins such as ADAM8, ADAM9, ADAM10, ADAM12, ADAM15, ADAM17, ADAM19, ADAM28 are overexpressed in human malignant tumors. ADAM proteins participate in mediating ectodomain-shedding of several proteins, including tumor necrosis factor-*α* (TNF), transforming growth factor (TGF)-*α* and heparin-binding-epidermal growth factor (HB-EGF) [43, 45, 46]. Dysregulation of TNF production has been linked to a variety of human diseases including Alzheimer’s disease, major depression and cancer [47]. Up to now, several pathways have been postulated to account for the mediation of ADAM in cancer cell proliferation and progression. One of them is the ectodomain-shedding of growth factors TGF-*α* and HB-EGF. This process perhaps alter signaling on the surfaces of cancer cells, inducing amplified cell proliferation through autocrine and paracrine mechanism [43]. According to our data, we speculate that like many of the *ADAM*s, *ADAM32* may have importance in cancer cell proliferation and progression.

29% of the set of NOF-BPRs were classified as cancer-specific. The gene *RP11-115C21.2* (chr8: 6,261,072 bp to 6,264,663 bp), coding for a large intergenic non-coding RNA (lincRNA), is localized close to one BPR in over 75% of the analyzed tumor entities (≥ 1% occurrence). In 5 tumor entities, the region of the gene was affected by an extended deletion. The lincRNAs make up the most of the long non-coding RNAs (lncRNAs). In the last few years, the importance of the lncRNA has been uncovered for tumorigenesis and mutagenesis. LncRNA can appear as either oncogene or tumor suppressor gene by alteration in the structure of chromatin [48–50] and also affect the transcription of protein-coding genes [51]. Based on our analyses, it could be suggested that the regulation of cell cycle and apoptosis were disturbed because of the deleted segments and the *RP11-115C21.2* gene operated as tumor suppressor gene.

By contrast, only 16% of the NOF-BPRs were found to be tumor entity-specific. This allows for the assumption that higher entity-specific BPRs could be detected preferably in multiple tumor entities. A similar idea has also been proposed by a study of somatic copy number alterations [35]. In this manner, it could be supposed that the genomic alterations, which promote a cancer disease, are common in multiple tumor entities. Only a few of individual alterations are associated with single tumor entities.

One out of this set of BPRs is located on chromosome 17 between 18,917,915 bp and 19,168,912 bp and was only detectable in brain tumor tissues with sufficient frequencies. By contrast, the complete region of 6,689 bp to 18,917,915 bp were deleted in three other tumor entities and in the brain cancer. In this section, several tumor-associated genes are coded, and therefore being affected. Among these, the most known gene coded for the tumor suppressor p53 (*TP53* 7,565,097 bp—7,590,856 bp) is important for cycle arrest, apoptosis, senescence, DNA repair and evokes changes in metabolism [52, 53].

## Conclusion

In conclusion, our analysis of genetic variations ties CNV detection, evaluation of BPR occurrence frequency, identification of CNV-affecting genes and functional annotation. By studying a large number of tumor and healthy samples of diverse tissue origins, we have found that BPRs tended to occur more frequently in certain genomic regions in the tumor samples whereas being genome-wide dispersed in the normal samples. In general, therefore, it seems that some regions are preferential targets for the underlying mutations, suggesting the non-randomness of CNV mutations in tumor genomes. The higher recurrency of the tumor entity-specific BPRs could also suggests that there are several tissue-specific mechanisms of tumorigenesis and progression. The strongly enhanced occurrence of specific BPRs in tumors may be due to increased cell proliferation; however, several known tumor-associated genes were colocalized in the same genomic regions, and thus supporting common progression mechanism that explains the increasing fragmentation of DNA along with tumor progression. A majority of the identified tumor-relevant BPRs are either tumor entity-specific or associated with multiple-entities (termed ‘cancer-specific’). A small part of these BPRs (termed ‘common BPRs’) exhibited also a noticeable occurrence frequency in the normal samples. Further we observed hotspots at which segments with similar alterations in copy number were overlapped along with BPRs adjacently clustered. The presence of those hotspots and common BPRs imply that frequently affected mutations at fragile sites loci might also be responsible for cancer-gene alteration. Colocalization assessment and functional annotation revealed that not only protein-coding genes but also long intergenic non-coding RNAs were affected by CNV genomic regions, suggesting that both protein-coding and non-coding genes with a broad range of biological functions might play a causative or functional role in tumor biology. The findings of the present study with larger sets of samples of diverse tissue origins would serve as viable clues to the interpretation of the mechanisms for carcinogenesis.

Identifying BPRs and characterizing their influence on tumor phenotypes can help to identify molecular factors and biomarkers responsible for tumorigenesis and progression, and hence developing new and effective therapeutic strategies. The sorting of BPRs into BPR recurrency classes would be a suitable starting point. We have found a set of BPRs with noticeable occurrence frequency in specific tumor entities. However, more research on this topic needs to be undertaken before the asscociation between BPR classification and tumor phenotypes is more rationally established. This study showed that tumor genomes exhibit a large set of BPRs. There are many factors contributing to DNA breakpoints, for example, increased cell proliferation, failed DNA repair mechanism, or vulnerability to specific processes or damages and histone modifications. In particular, interest in histone modifications has grown over the last decade because alterations in the function of histone-modifying lead to oncogenic transformation. With the methods in the present study, however, it is not possible to address the influence of this causual variant on cancer development and progression. Note that genes interact with other genes in complex signaling or regulatory networks, and pathways are more likely to cooperate together, it would be desirable to incorporate information about different pathways possibly involved in cancer cell proliferation and progression.

## Supporting Information

S1 Table Tumor Samples. List of GEO accession (GSM) numbers for all used tumor samples.
(CSV)

S2 Table Reference Samples. List of all used sample names from HapMap Project (Phase 3, Release #3) for the pooled reference dataset.
(TXT)

S3 Table Normal Samples. List of GEO accession (GSM) numbers for all used samples from healthy tissue.
(CSV)

S4 Table Overview over all detected breakpoint regions (BPRs) (tumor and healthy tissue samples). Data presented here include the related chromosome, start and end positions, relative occurrence probability in the particular tissues, the total occurrence over all samples and the related classification of the BPR.
(CSV)

S5 Table Segments of copy number alteration in brain cancer (pediatric medulloblastoma). Averaged *log*_2_ ratios copy number alterations were evaluated for brain cancer tissues. Additional information presented in the table includes chromosome number, types of alterations (deletion—loss, amplification -gain), start and end positions.
(TXT)

S6 Table Segments with altered copy numbers in breast cancer. Altered averaged *log*_2_ ratios from chromosomal average in breast cancer tissues were given, together with chromosome number, types of alterations (deletion—loss, amplification -gain), start and end positions.
(TXT)

S7 Table Segments with altered copy numbers in colorectal cancer. Averaged *log*_2_ ratios from chromosomal average in colorectal cancer tissues are presented, together with additional information including chromosome number, types of alterations (deletion—loss, amplification -gain), start and end positions.
(TXT)

S8 Table Segments with altered copy numbers in gastric cancer. Averaged *log*_2_ ratios from chromosomal average in gastric cancer tissues were calculated. Additional results obtained include chromosome number, types of alterations (deletion—loss, amplification -gain), start and end positions.
(TXT)

S9 Table Segments with altered copy numbers in lung cancer. Averaged *log*_2_ ratios from chromosomal average in lung cancer tissues are given together with information including chromosome number, types of alterations (deletion—loss, amplification -gain), start and end positions.
(TXT)

S10 Table Segments with altered copy numbers in ovarian cancer. Averaged *log*_2_ ratios from chromosomal average in ovarian cancer tissue are presented together with additioinal information including chromosome number, types of alterations (deletion—loss, amplification -gain), start and end positions.
(TXT)

S11 Table Segments with altered copy numbers in prostate cancer. Averaged *log*_2_ ratios from chromosomal average in prostate cancer tissues are given, together with additioinal information including chromosome number, types of alterations (deletion—loss, amplification -gain), start and end positions.
(TXT)

S12 Table Segments with altered copy numbers in renal cancer. Averaged *log*_2_ ratios from chromosomal average in renal cancer tissues are shown together with additional information including chromosome number, types of alterations (deletion—loss, amplification -gain), start and end positions.
(TXT)

S13 Table Segments with altered copy numbers in healthy tissues. Data presented here are *log*_2_ ratios from chromosomal average in healthy tissues, chromosome number, types of alterations (deletion—loss, amplification—gain), start and end position.
(TXT)

S14 Table Overview over all detected potentially affected genes due to segments with altered copy number. For the potentially affected genes, Ensembl IDs are given together with additional information, including gene names (if known), the related chromosome, the start and end positions of the genes, whether a gene is affected at least 1 time in the respective tissue (1—0 if not) and the total number of potentially affected tissues.
(CSV)

S15 Table Potentially affected tumor-associated genes. Only those genes which are known as tumor-associated due to segments with altered copy number were summarized. The affected genes are demonstrated with the Ensembl IDs, gene names (if known), the related chromosome, the start and end positions of the genes, whether a gene is affected at least 1 time in the respective tissue (1—0 if not) and the total number of potentially affected tissues.
(CSV)

S16 Table Potentially affected genes in the healthy tissues. Only those genes which have segments of altered copy number in healthy tissues were summarized. The affected genes are demonstrated with the Ensembl IDs, gene name, the related chromosome, the start and end positions of the genes, whether a gene is affected at least 1 time in the respective tissue (1—0 if not) and the total number of potentially affected tissues.
(CSV)

S1 Fig Circos plot of copy number changes over the full genome. Averaged *log*_2_ ratios of copy number were evaluated for the tumor entities and the healthy tissue samples over all autosomal chromosomes. Data are shown with entities numbered (1- brain cancer pediatric medulloblastoma, 2- breast cancer, 3- colorectal cancer, 4- gastric cancer, 5- lung cancer, 6- ovarian cancer, 7- prostate cancer, 8- renal cancer, 9- healthy tissues).
(PDF)

S2 Fig Circos plot of copy number changes in chromosome 16. Averaged *log*_2_-ratios of the tumor entities and the healthy tissue samples in single plots over chromosome 16 are presented with entities numbered (1- brain cancer pediatric medulloblastoma, 2- breast cancer, 3- colorectal cancer, 4- gastric cancer, 5- lung cancer, 6- ovarian cancer, 7- prostate cancer, 8- renal cancer, 9- healthy tissues).
(PDF)

S3 Fig Circos plot of copy number changes in chromosome 17. Averaged *log*_2_-ratios of the tumor entities and the healthy tissue samples over chromosome 17 are shown with entities numbered (1- brain cancer pediatric medulloblastoma, 2- breast cancer, 3- colorectal cancer, 4- gastric cancer, 5- lung cancer, 6- ovarian cancer, 7- prostate cancer, 8- renal cancer, 9- healthy tissues).
(PDF)

S4 Fig Bar plot of the occurrence of BPRs within genomic and intergenic regions. The numbers of intragenic and intergenic BPRs are shown as percentages of intragenic (dark green) regions, regions which are overlapping intra- and intergenic regions (light green) and intergenic region (grey) for every tumor entity and the healthy tissue.
(PDF)

S5 Fig Bar plot of the occurrence of segments of altered copy number within intragenic and intergenic regions. The number of intragenic and intergenic segments of altered copy number were counted. It is shown the percentages of intragenic (dark green) regions, regions which are overlapping intra- and intergenic regions (light green) and intergenic region (grey) for every tumor entity and the healthy tissue.
(PDF)

S6 Fig Bar plot of the frequency of possibly affected genes by segments of altered copy number. The following figure illustrates the number of affected genes (grey) compared to the number of affected tumor associated genes (green) for every tumor entity and the healthy tissue.
(PDF)

S7 Fig Number of breakpoints per sample. The sorted numbers of breakpoints identifed per sample are plotted. The abscissa gives the index of the sample and the ordinate the counted breakpoints. The samples are grouped in tissue types.
(PDF)
