# Supplementary figures and images for: Comprehensive Analysis of Genome Rearrangements in Eight Human Malignant Tumor Tissues
Source: PLoS One. 2016 Jul 8;11(7):e0158995. doi: 10.1371/journal.pone.0158995 (PMC4938598; doi:10.1371/journal.pone.0158995)

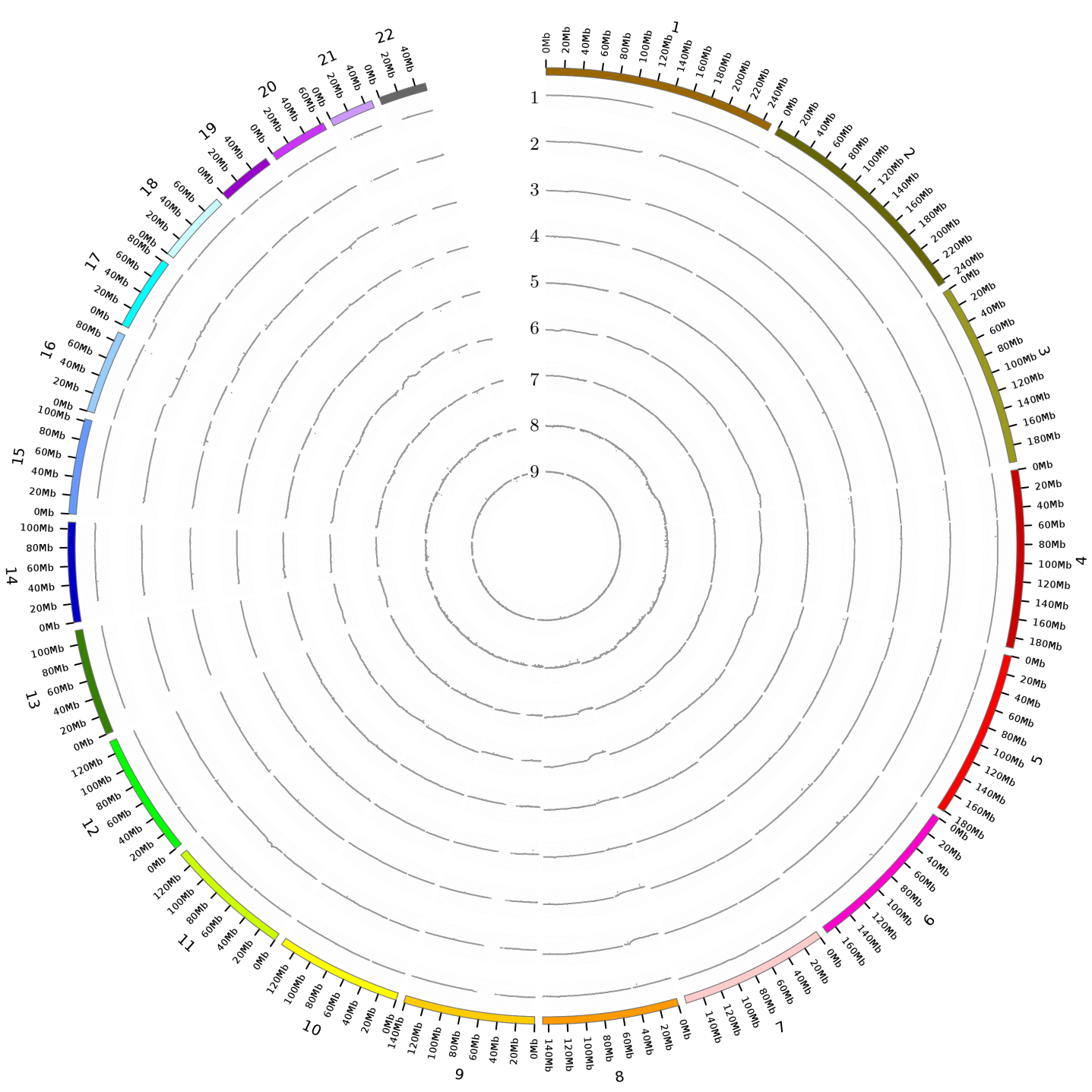

Supplement: S1 Fig — Averaged log2 ratios of copy number were evaluated for the tumor entities and the healthy tissue samples over all autosomal chromosomes. Data are shown with entities numbered (1- brain cancer pediatric medulloblastoma, 2- breast cancer, 3- colorectal cancer, 4- gastric cancer, 5- lung cancer, 6- ovarian cancer, 7- prostate cancer, 8- renal cancer, 9- healthy tissues). (PDF) [file pone.0158995.s017.pdf]

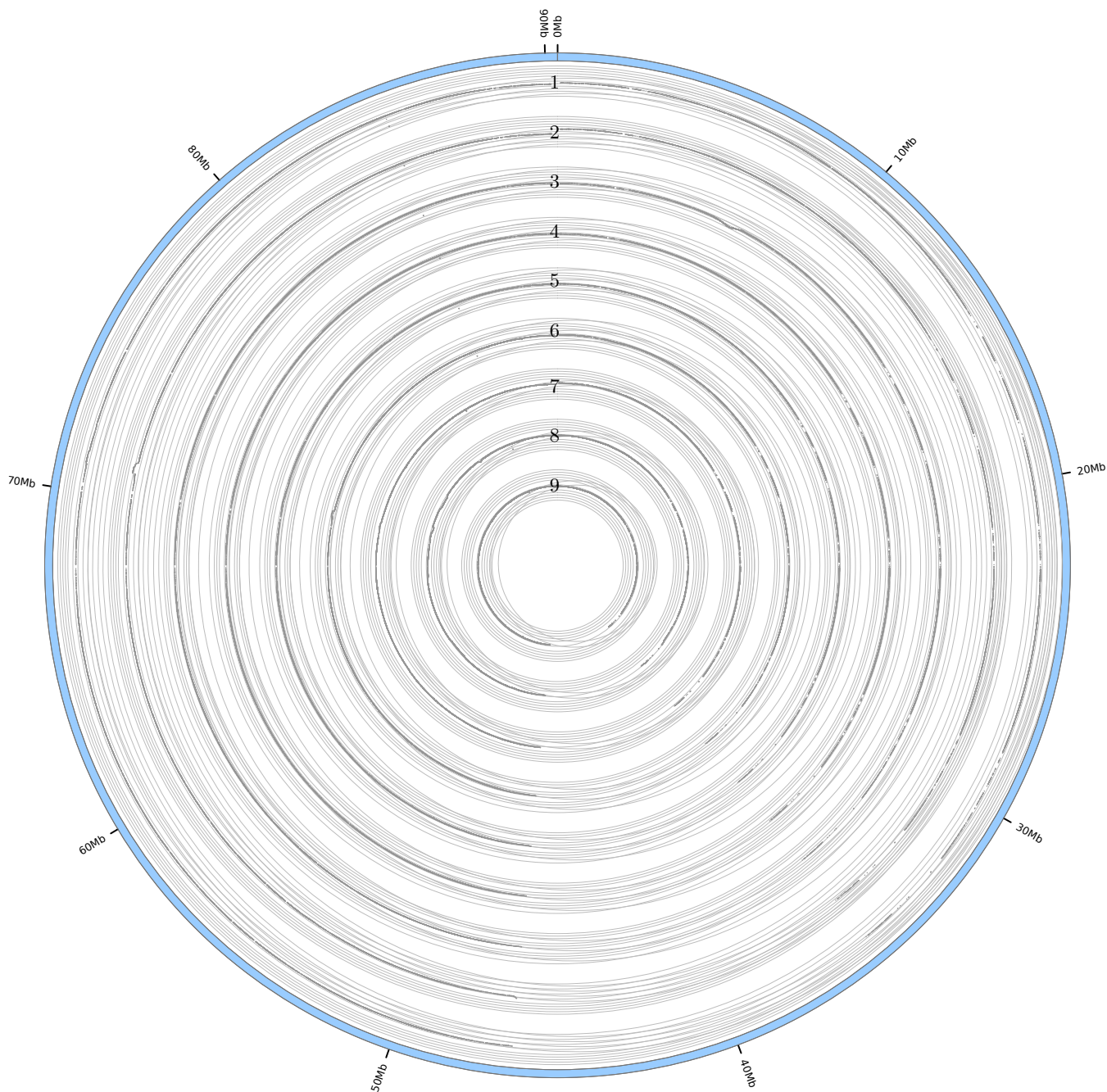

Supplement: S2 Fig — Averaged log2-ratios of the tumor entities and the healthy tissue samples in single plots over chromosome 16 are presented with entities numbered (1- brain cancer pediatric medulloblastoma, 2- breast cancer, 3- colorectal cancer, 4- gastric cancer, 5- lung cancer, 6- ovarian cancer, 7- prostate cancer, 8- renal cancer, 9- healthy tissues). (PDF) [file pone.0158995.s018.pdf]

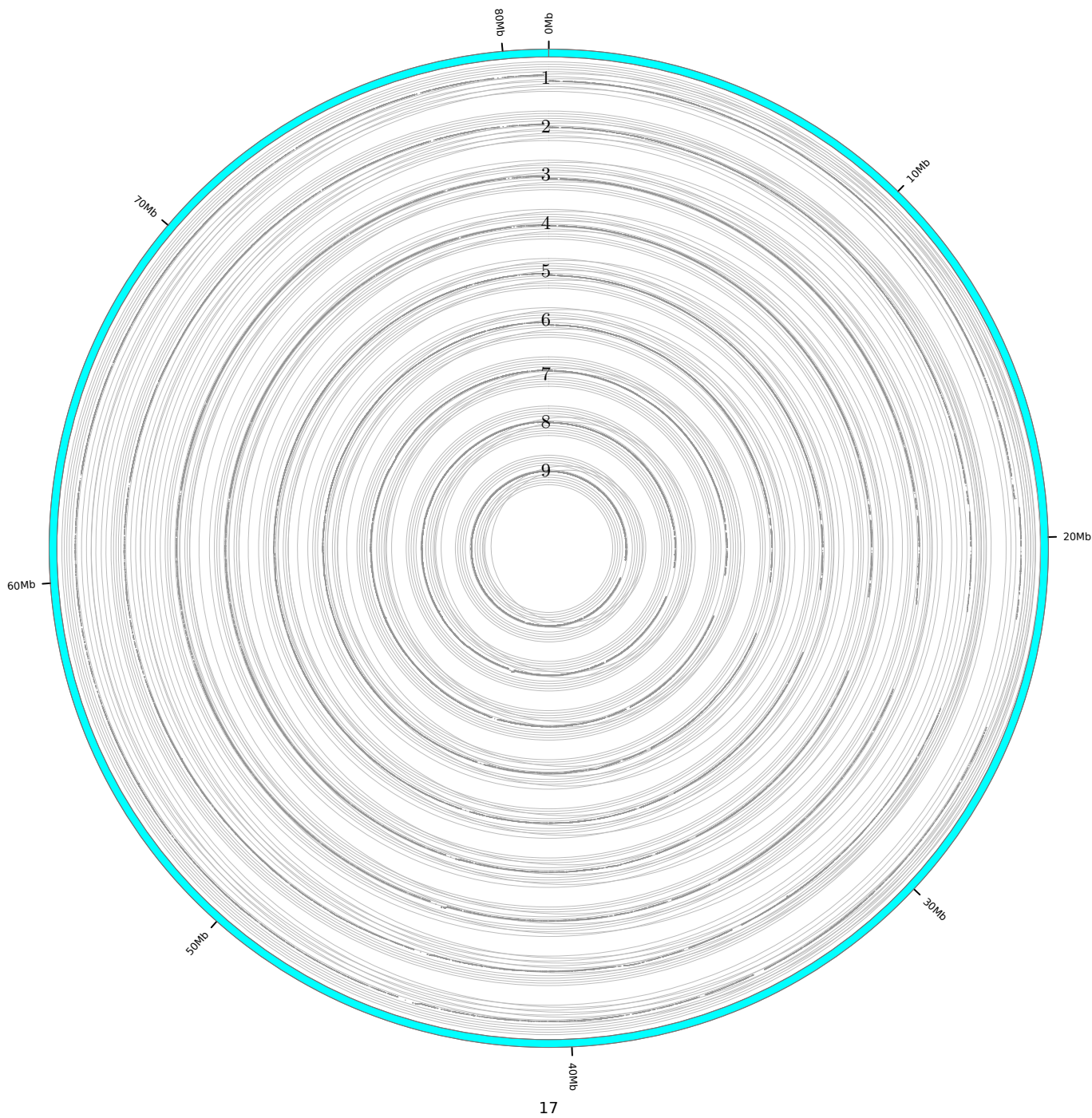

Supplement: S3 Fig — Averaged log2-ratios of the tumor entities and the healthy tissue samples over chromosome 17 are shown with entities numbered (1- brain cancer pediatric medulloblastoma, 2- breast cancer, 3- colorectal cancer, 4- gastric cancer, 5- lung cancer, 6- ovarian cancer, 7- prostate cancer, 8- renal cancer, 9- healthy tissues). (PDF) [file pone.0158995.s019.pdf]

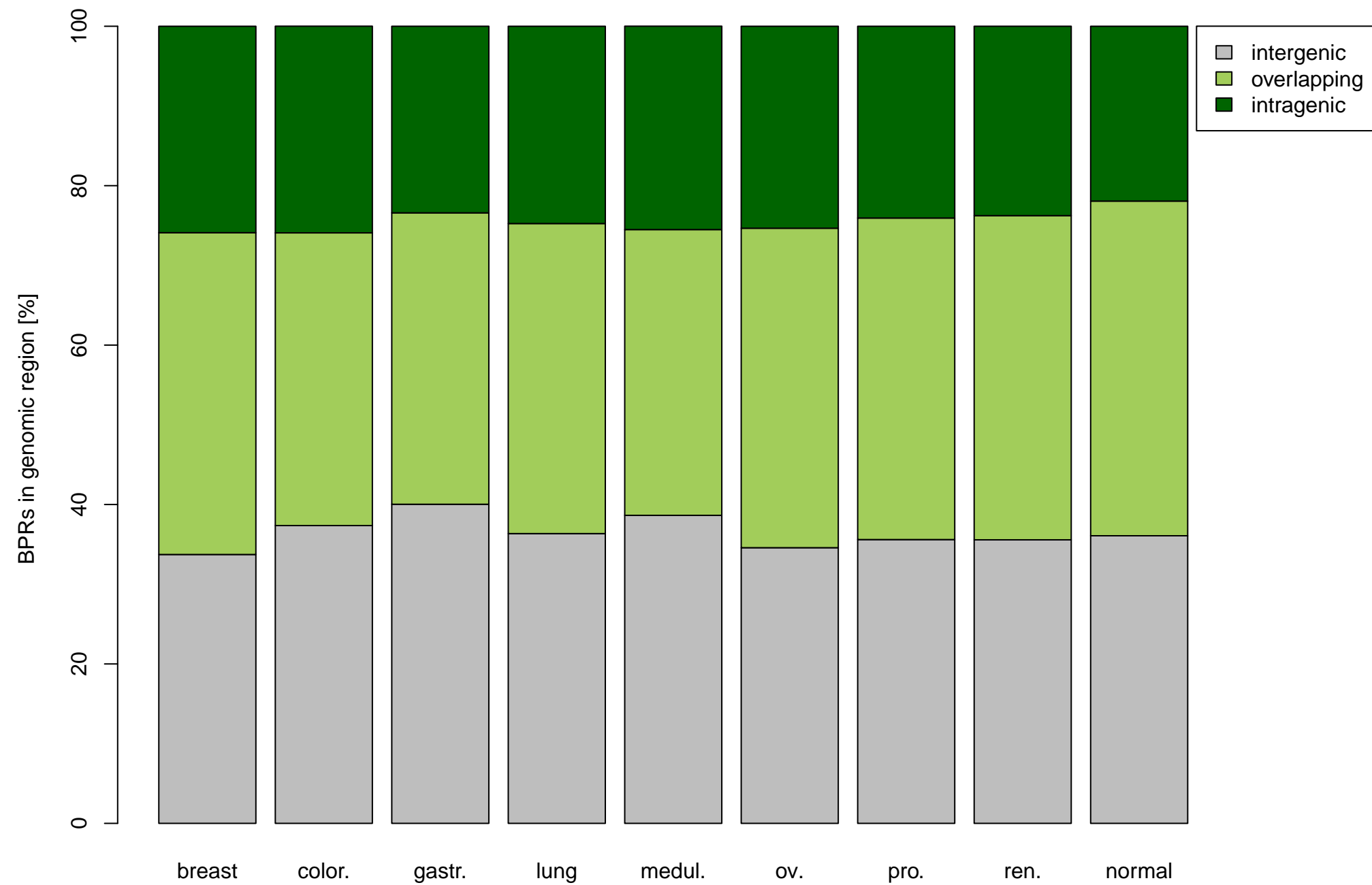

Supplement: S4 Fig — The numbers of intragenic and intergenic BPRs are shown as percentages of intragenic (dark green) regions, regions which are overlapping intra- and intergenic regions (light green) and intergenic region (grey) for every tumor entity and the healthy tissue. (PDF) [file pone.0158995.s020.pdf]

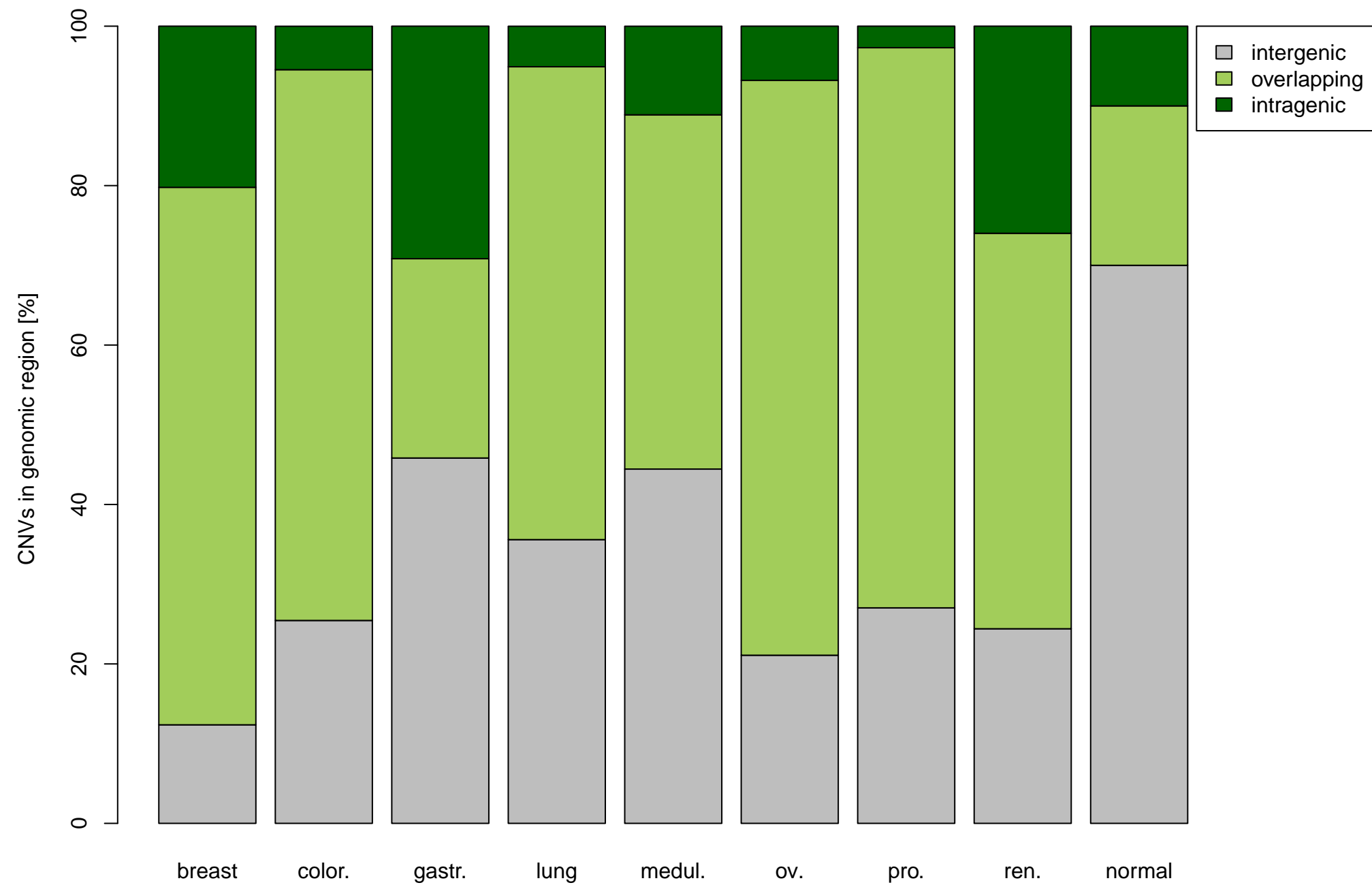

Supplement: S5 Fig — The number of intragenic and intergenic segments of altered copy number were counted. It is shown the percentages of intragenic (dark green) regions, regions which are overlapping intra- and intergenic regions (light green) and intergenic region (grey) for every tumor entity and the healthy tissue. (PDF) [file pone.0158995.s021.pdf]

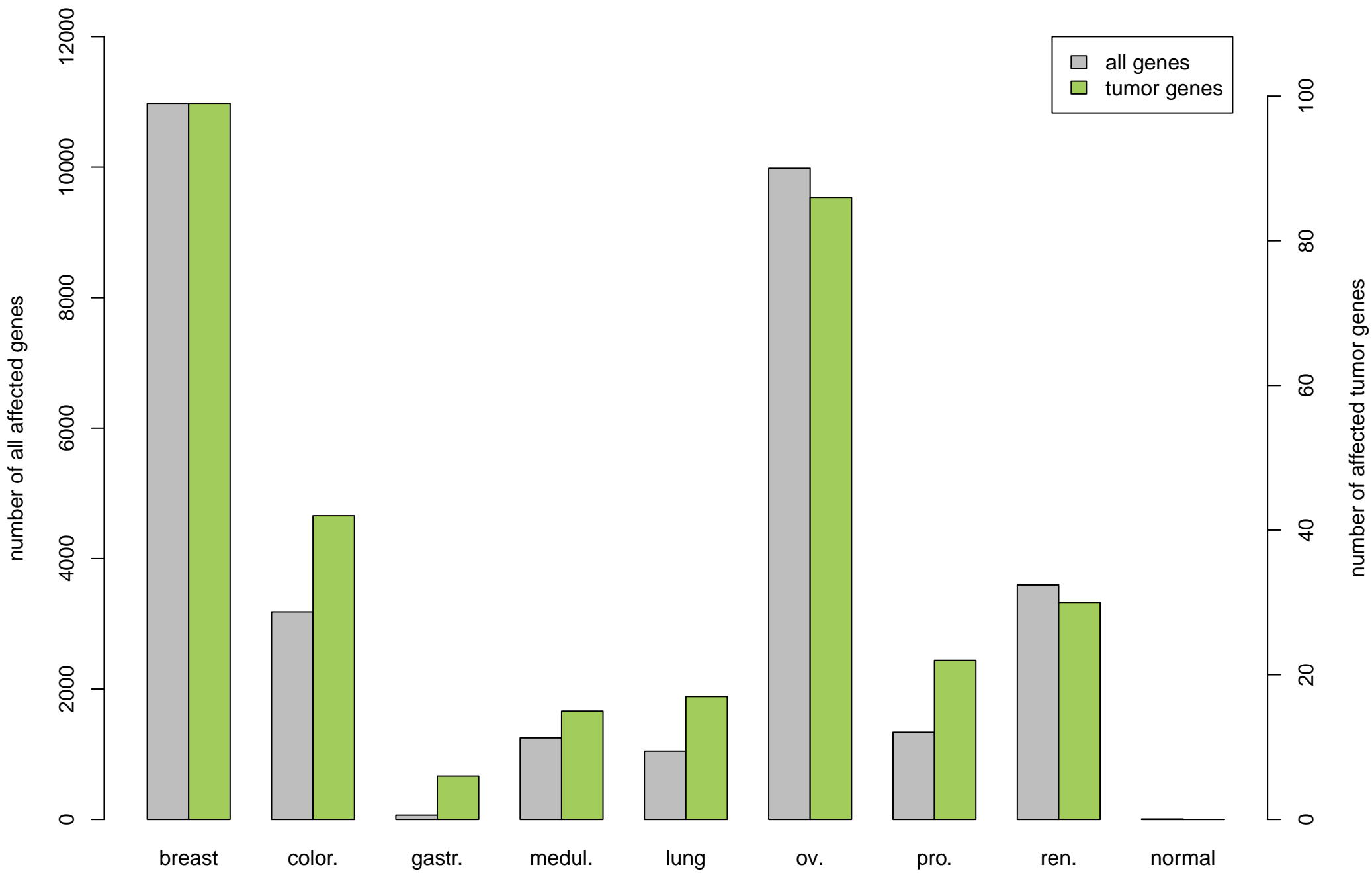

Supplement: S6 Fig — The following figure illustrates the number of affected genes (grey) compared to the number of affected tumor associated genes (green) for every tumor entity and the healthy tissue. (PDF) [file pone.0158995.s022.pdf]

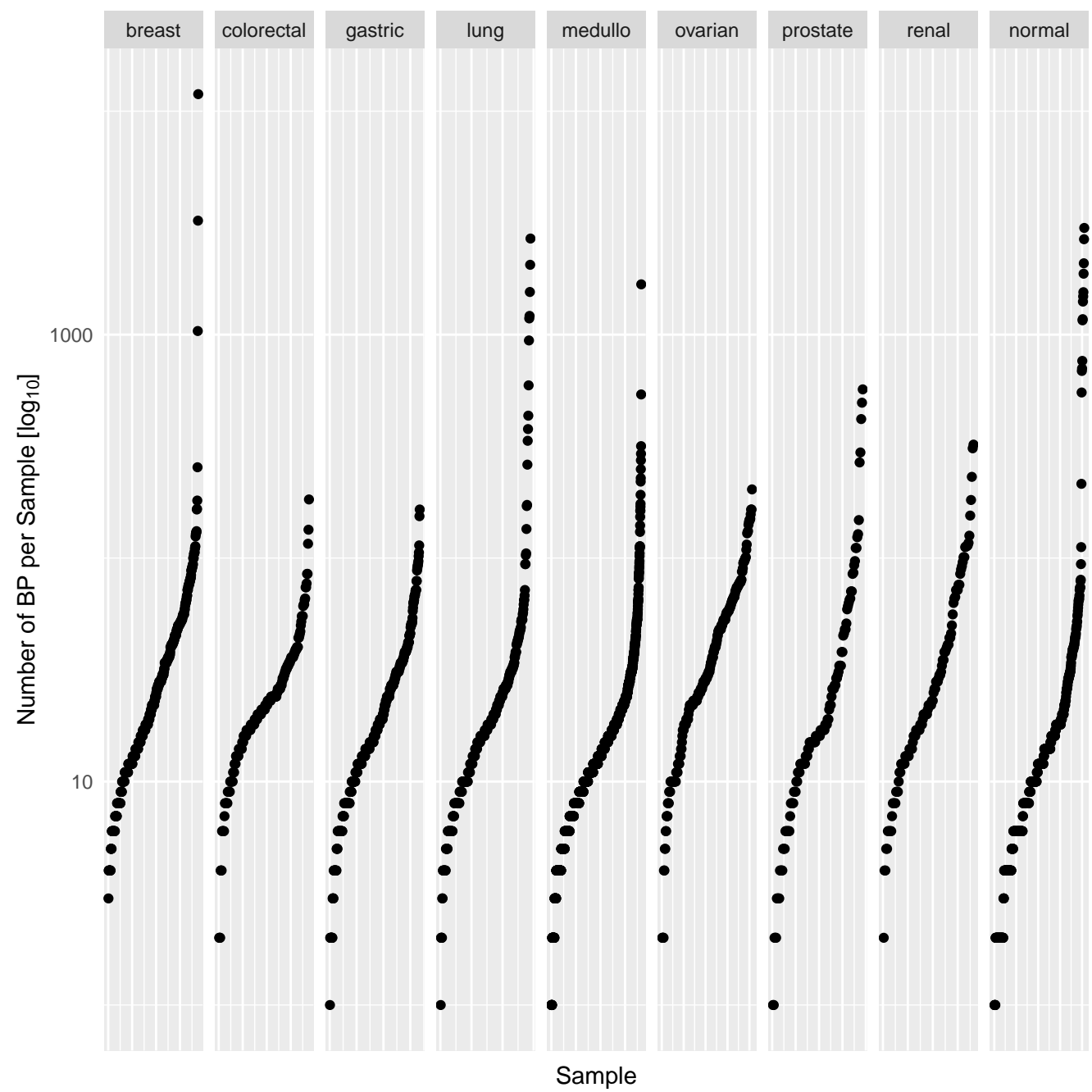

Supplement: S7 Fig — The sorted numbers of breakpoints identifed per sample are plotted. The abscissa gives the index of the sample and the ordinate the counted breakpoints. The samples are grouped in tissue types. (PDF) [file pone.0158995.s023.pdf]
